# Reasons for home delivery and use of traditional birth attendants in rural Zambia: a qualitative study

**DOI:** 10.1186/s12884-015-0652-7

**Published:** 2015-09-11

**Authors:** Cephas Sialubanje, Karlijn Massar, Davidson H. Hamer, Robert AC Ruiter

**Affiliations:** Ministry of Health, Monze District Medical Office, P.O. Box 660144, Monze, Zambia; Department of Work and Social Psychology, Maastricht University, P.O. Box 616, , 6200MD Maastricht, The Netherlands; Zambia Centre for Applied Health Research and Development, P.O. Box 30910, Lusaka, Zambia; Centre for Global Health and Development Boston University, Crosstown 3rd floor, 801 Massachusetts Avenue, Boston, MA 02118 USA; Department of International Health, Boston University School of Public Health, Crosstown 3rd floor, 801 Massachusetts Avenue, Boston, MA 02118 USA

## Abstract

**Background:**

Despite the policy change stopping traditional birth attendants (TBAs) from conducting deliveries at home and encouraging all women to give birth at the clinic under skilled care, many women still give birth at home and TBAs are essential providers of obstetric care in rural Zambia. The main reasons for pregnant women’s preference for TBAs are not well understood. This qualitative study aimed to identify reasons motivating women to giving birth at home and seek the help of TBAs. This knowledge is important for the design of public health interventions focusing on promoting facility-based skilled birth attendance in Zambia.

**Methods:**

We conducted ten focus group discussions (*n* = 100) with women of reproductive age (15–45 years) in five health centre catchment areas with the lowest institutional delivery rates in the district. In addition, a total of 30 in-depth interviews were conducted comprising 5 TBAs, 4 headmen, 4 husbands, 4 mothers, 4 neighbourhood health committee (NHC) members, 4 community health workers (CHWs) and 5 nurses. Perspectives on TBAs, the decision-making process regarding home delivery and use of TBAs, and reasons for preference of TBAs and their services were explored.

**Results:**

Our findings show that women’s lack of decision- making autonomy regarding child birth, dependence on the husband and other family members for the final decision, and various physical and socioeconomic barriers including long distances, lack of money for transport and the requirement to bring baby clothes and food while staying at the clinic, prevented them from delivering at a clinic. In addition, socio-cultural norms regarding childbirth, negative attitude towards the quality of services provided at the clinic, made most women deliver at home. Moreover, most women had a positive attitude towards TBAs and perceived them to be respectful, skilled, friendly, trustworthy, and available when they needed them.

**Conclusion:**

Our findings suggest a need to empower women with decision-making skills regarding childbirth and to lower barriers that prevent them from going to the health facility in time. There is also need to improve the quality of existing facility-based delivery services and to strengthen linkages between TBAs and the formal health system.

## Background

Zambia is one of the sub-Saharan African countries with a high maternal mortality ratio (MMR) [1, 2]. The latest demographic and health survey (DHS) [1] showed that the country’s MMR is 591 maternal deaths per 100 000 live births. Moreover, more than half (53 %) of the women in Zambia, do not receive skilled birth attendance, The survey [1] further shows that these numbers are even higher in rural areas where more than seventy percent of the women give birth at home, outside the health facility, and are often assisted by TBAs [1].

The World Health Organisation (WHO) has defined TBAs as persons who assist the mother during childbirth and learns her skills through apprenticeship that involves both observation and imitation, and is often highly regarded by the community that chooses her to assist women in childbirth [3]. Reviews [4] and studies conducted in Zambia [5] and other developing countries [6, 7] have reported the effectiveness of TBAs in improving maternal and newborn health outcomes. For example Gill and colleagues [5] showed that training TBAs to manage common perinatal conditions significantly reduced neonatal mortality in Lufwanya, Zambia.

Although training TBAs may provide them with basic midwifery skills, most TBAs have no access to the requisite clean delivery tools such as supply of drugs and equipment for obstetric care [5]; this may increase the risk for infections during childbirth. Moreover, the TBAs have no access to referral services to the hospital in case of complications during and after labour [8].

Consequently, there has been a policy change in many developing countries − including Zambia [9] to stop the funding and training of TBA programmes. Rather, all women are recommended to use facility-based delivery services provided by trained and skilled healthcare staff [8]. This change in policy has resulted in TBAs not being recognised as part of the providers of essential obstetric care in Zambia [9]. Nevertheless, many women in rural Zambia, still give birth at home and TBAs are essential providers of obstetric care [1, 10, 11].

There is a lack of evidence on the main reasons for home delivery and use of TBAs. Currently, most published studies investigating reasons for home delivery in Zambia [12, 13] and other developing countries [14–16] have focused on the structural barriers to facility delivery services such as lack of geographical access to emergency obstetric care and financial limitations. Factors that motivate women to give birth at home and their reasons to seek the assistance of TBAs have not been explored in depth. One qualitative study conducted in Zambia [10] showed that perceived poor quality of maternal healthcare services (MHS) due to negative staff attitudes towards pregnant women, a shortage of qualified staff and a lack of drugs and supplies necessary for emergency obstetric care, social and cultural norms, women’s low social status and lack of decision-making autonomy prevented women from utilising facility delivery services in rural Zambia. Similarly, Titaley [17] and colleagues in Indonesia showed that the community had positive attitude towards TBAs and perceived their role as essential for providing MHS. Women preferred services of the TBAs because they were available and easily accessible, affordable and pragmatic. Moreover, the community believed that TBAs had enough midwifery knowledge, skills, and that they were trustworthy. However, these factors may differ from one socioeconomic, cultural and geographic context to another, and may not be applicable to Zambia. Thus far, however, no studies have explored factors that influence women’s decision to give birth at home in rural Zambia, and reasons to seek the assistance of TBAs are not yet explored in-depth.

The purpose of this study, therefore, is to gain a better insight into women’s reasons to give birth at home and their preference for TBAs. Knowledge about these factors is important for the design of public health interventions focusing on promoting institutional deliveries and ultimately, improving maternal and newborn health outcomes in Zambia.

## Methods

### Study area

This qualitative study was conducted in Kalomo district, Zambia (for details on the district profile see Sialubanje et al. [10]) and used focus group discussions (FGDs) and in-depth interviews (IDIs) as data collection methods. We conducted both FGDs and IDIs in order to provide a detailed understanding of the factors under investigation. The Tropical Diseases Research Centre Ethics Review Committee and the Ministry of Health Research and Ethics Committee in Zambia granted ethical approval.

### Study population and sampling

Study participants were purposefully selected from five rural health centre (RHC) catchment areas with the lowest institutional delivery rates in the district, and comprised 100 women of reproductive age (15 to 45 years) who had given birth within one year prior to the study. These participated in the FGDs. In addition, the study included 30 IDI participants comprising 5 TBAs, 5 nurse-midwives, 4 women, 4 husbands, 4 headmen, 4 NHCs, and 4 CHWs aged between 20 and 45 years.

Selection of participants and study area (villages) was done in three stages using a purposeful homogeneous sampling technique which focuses on a subpopulation and area in which the respondents share similar characteristics. This sampling technique, in turn, helps the researcher to understand and describe something about a particular group in depth [18, 19]. In addition, this sampling technique allowed for selection of respondents with similar experience regarding childbirth and utilisation of MHS, while, at the same time, allowing for recruitment of respondents with different characteristics in terms of their age, number of children, marital status, and education level, which helped provide insight into the similarities and differences in their experiences.

To begin with, six RHCs with the lowest MHS utilisation rates in the district were purposefully selected with assistance from the District Medical Office. Next, two villages from the list of villages in each RHC catchment area were purposefully selected in consultation with local health centre staff and NHC members, giving a total of twelve villages. Village headmen and NHC members from the selected villages were then informed about the study and were requested to communicate the study and its purpose to their community members. This was done through the routine community meetings. Community members who were willing to participate in the study were requested to come to an agreed-upon place (normally the home of the headman) in the village for the interview on a date set by the research team.

All the women of reproductive age, aged between 15 and 45 years within the selected villages, and who had given birth one year prior to the survey, were eligible to participate in the FGD. Women aged below 15 and above 45 years were excluded from participation. In addition, women who had resided in the area for less than six months were also excluded because the investigators thought these women would not have had enough local experience on maternal health challenges and available resources.

A total of ten (10) FGDs were conducted, two in each health centre catchment area, one per village. After these 10 focus group discussions, data saturation was achieved, and two additional planned FGDs were not conducted.

In addition, to further obtain perspectives and experiences regarding the issue under investigation, a total of 30 IDIs with individuals assumed to represent the different subgroups and positions in the community were conducted. The IDIs comprised 5 TBAs, 4 headmen, 4 NHC members, 4 CHWs, 4 husbands, 4 mothers and 5 nurse-midwives. We purposefully selected three (3) key informants from each of the ten (10) villages under the five RHC catchment areas included in the study.

To be eligible to participate in the study, IDI participants should have been aged between 20 and 45 years. In addition, participants should have had resided in the area for more than six months; those who had resided in the area for less than six months were also excluded because of the reasons stated above.

### Data collection

Both FGDs and IDIs were conducted in the community by two trained research assistants. One research assistant facilitated/conducted the FGD/ interview, while the second one recorded the session using a digital voice recorder. Both FGDs and IDIs were conducted in the local language, Tonga. To ensure privacy and confidentiality, each FGD and IDI was conducted in a quiet place, normally under a tree. .

Before each FGD and interview, written consent was obtained from participants by requesting them to read and sign the consent form, which was translated into Tonga. Next, each respondent was asked to complete a short demographic questionnaire. For those who could not read, research assistants read the consent form and the questionnaire and filled it in. We did not obtain prior parental consent for the minors included in the study because we had obtained ethical approval for the study from the Tropical Diseases Research Centre (TDRC) Ethics Review Committee and the Ministry of Health Research and Ethics Committee (MoH REC) in Zambia. Both bodies granted ethical approval for the respondents aged between 15–17 years who had given birth one year prior to the study to participate in the FGDs without explicit parental consent. This approval was based on the fact that women who are married or are emancipated have the right to make their own decisions under the Zambian customary law, and this includes those aged 15 years and older. Moreover, the FGD participants were recruited by the village headmen who are considered as consent-providers under the Zambian customary law. Furthermore, both in the recruitment and during the FGDs, it was stressed that participation was completely voluntary and that participants’ answers would be treated confidentially and anonymously.

A semi-structured interview guide translated into Tonga, was used for both FGDs and IDIs. The main topics explored included perspectives on home delivery and use of TBAs, decision-making processes regarding use of MHS, and reasons for home delivery and use of TBAs. IDI participants were mainly asked to share their experiences regarding the topic under discussion and provide more insight into the subject.

Each FGD was conducted in a quiet place in each village and lasted between one 1 and 1.5 h. In order to allow for free discussions among the participants, the FGDs were arranged into two groups, according to age: women aged between 15 and 19 years and those aged above 20 years. Each FGD consisted of 8 to 12 participants.

### Data analysis

All voice recordings from FGDs and interviews were transcribed and translated into English by research assistants. To check for accuracy, some (20 %) of the transcripts were back translated into Tonga. Members of the research team then compared the Tonga and English versions for differences and similarities while listening to the original voice recording. After verification of accuracy in translation, each transcript was then read aloud by a research assistant while the other team members were listening to the corresponding voice recording. Next, each translated transcript was compared with the hand-written field notes that the research assistants had prepared during the FGDs and interviews. After proof-reading and making corrections, the transcripts for both FGDs and IDIs were saved on a password-protected computer. The word documents were then exported into QSR (NVivo 9.0 software) for processing.

An inductive approach to data analysis was used, guided by three questions based on the three pre-determined themes: 1) what are the women’s perspectives on home delivery and use of TBAs? 2) how is the decision on the place of delivery made?, and 3) which factors influence the decision to give birth at home and use TBAs? In order to determine similarities and differences in the responses, findings for the FGDs and IDIs were analysed separately. We computed percentages of respondents’ demographic characteristics using IBM SPSS Statistics version 21 and the results are shown in Table 1 below.

**Table 1:** Background characteristics for the respondents

Characteristic	IDIs (*n* = 30)	*n* (%)	FGDs (*n* = 100)	*n* (%)
Age in years				
15–19		0 (0 %)		35 (35 %)
20–34		10 (33.33 %)		50 (50 %)
35 -45		12 (40 %)		15 (15 %)
Above 45		8 (26.67 %)		
Sex:				
Male		14 (46.67 %)		0 (0 %)
Female		16 (53.33 %)		100 (100 %)
Marital Status:				
Single		2 (6.67 %)		15 (15 %)
Married		28 (93.33 %)		70 (70 %)
Divorced		0 (0 %)		5 (5 %)
Widow		0 (0 %)		10 (10 %)
Number of children				
1–2		3 (10 %)		25 (25 %)
3–5		12 (40 %)		50 (50 %)
6 and above		15 (50 %)		25 (25 %)
Cadre				
TBA		5 (16.75 %)		
Village headman		4 (13.3 %)		
NHC members		4 (13.3 %)		
CHWs		4 (13.3 %)		
Husbands		4 (13.3 %)		
Women		4 (13.3 %)		
Nurse-midwives		5 (16.75 %)		
Level of education:				
Never attended school		0 (0 %)		7 (7 %)
Lower primary (1–4)		0 (0 %)		10 (10 %)
Upper primary (5–7)		15 (50 %)		48 (48 %)
Junior secondary (8–9)		6 (20 %)		25 (25 %)
Senior Secondary (10–12)		4 (13.3 %)		10 (10 %)
Tertiary		5(16.7 %)		0 (0 %)
Occupation:				
Unemployed		0 (0 %)		15 (15 %)
Farmer		25 (83.3 %)		75 (75 %)
Self- employed		0 (0 %)		0 (0 %)
Formal employment		5 (16.7 %)		10 (10 %)
Level of income:				
<$20		15(50 %)		65 (65 %)
$20–49		10 (33.3 %)		25 (25 %)
$50–99		0 (%)		10 (10 %)
$100–199		0 (%)		0 (0 %)
>200		5 (16.7 %)		0 (0 %)
Place of delivery for the youngest child				
Clinic		3 out of 4 mothers (75 %)		47(47 %)
Home		1 out of 4 mothers (25 %)		53 (53 %)
On the way to the clinic		0 (0 %)		0 (0 %)

### Ethical approval

Ethical approval was obtained from the Tropical Diseases Research Centre Ethics Review Committee and the Ministry of Health Research and Ethics Committee in Zambia.

## Results

### Demographics

A total of 130 respondents participated in the study. Of the 100 FGD participants, 35 (35 %) were aged between 15 and 19 years, 50 (50 %) between 20 and 34 years, and 15 (15 %) were aged between 35 and 45 years. Of the 30 key informants, 10 (33.33 %) were aged between 20 and 34 years, 12 (40 %) were aged between 35 and 45 years, and 8 (26.67 %) were aged above 45 years. All the FGD respondents were female, while 16 (53.33 %) of the IDI respondents were female and 14 (46.67 %) were male. The majority (73.08 %) of the FGD respondents were married, 15 % were single, 10 % were widowed, and 5 % were divorced. The majority of the IDI respondents (93.33 %) were married and only 2 (6.67 %) were single and included 5 (16.75 %) TBAs, 5 (16.75 %) nurse-midwives, 4 (13.3 %) women, 4(13.3 %) husbands, 4 (13.3 %) headmen, 4 (13.3 %) NHCs members, and 4 (13.3 %) CHWs. Half (50 %) of the FGD respondents had between 3 and 5 children, 25 % of them had either 1 or 2 children, and the other 25 % had 6 or more children. Half (50 %) of the IDI respondents had between 6 and more children, 40 % had between 3 and 5 children and 10 % had either 1 or 2 children. About half (48 %) of the FGD respondents had upper primary school education, a quarter (25 %) had junior secondary school education, 10 % had senior secondary school education, 7 % had never attended school and 10 % had lower primary school education.

Half (50 %) of the IDI respondents had upper primary school education, 20 % had junior secondary school education, 13.3 % had senior secondary school education and 16.7 % had tertiary education. The majority (75 %) of FGD and IDIs (83.3 %) respondents were farmers and earned less than 20 US dollars per month, while 16.7 % of the IDI respondents were in formal employment and earned more than 200 US dollars per month. The majority (53 %) of the FGD respondents had given birth at home during their previous pregnancy, while 3 out of the 4 mothers (75 %) IDI respondents had given birth at the clinic (see Table 1).

### Theme 1: perspectives on home delivery and use of traditional birth attendants

This theme focused on IDI and FGD respondents’ perspectives on home delivery and use of TBAs and their services. Overall, all IDI and FGD respondents were aware of the TBAs in their communities. One IDI respondent described them as follows:

“*TBAs are women who live with us in the community. They were trained as nurses, but they are not nurses; they are women who were trained so that they help women from their villages during labour” (35 year old IDI respondent/ CHW).*

Moreover, most IDI and FGD respondents had a positive attitude towards TBAs and their services. Exceptions were the 75 % young FGD respondents and 25 % old FGD respondents with one or two children. Respondents who had a positive attitude towards TBAs explained that TBAs were very important and helpful because they assisted women during labour in their communities. In addition, TBAs played an important role in providing health education and antenatal care (ANC) services in designated health posts in the community. Furthermore, TBAs conducted deliveries in the women’s homes.

Moreover, all the five nurses participating in the IDIs explained that TBAs were still needed because of poor staffing levels in the rural areas. They revealed that in some RHCs, TBAs assisted nurses to provide ANC services and to conduct deliveries.

Concerning their training, four (4) out of the five (5) TBAs mentioned that they were trained by staff from the district medical office (DMO). They also explained that some of their colleagues were trained by other organisations such as World Vision International (WVI) and the Churches Health Association of Zambia (CHAZ). In contrast, one TBA mentioned that she was not trained. Furthermore, all the five argued that, although most of the TBAs in their communities were trained, some of them were not. Rather, they got the knowledge and skills from the old women in their communities.

*“We were trained by the Ministry of Health a long time ago. They trained us on how to examine the mother during pregnancy, conduct deliveries and protect the baby. Our training used to take six weeks. The people from the district used to train us, but now they have stopped” (45 year old traditional birth attendants).*

All the IDI and FGD participants were aware of the new policy that stopped the training of TBAs. They explained that the new policy also recommended that TBAs stop conducting deliveries and that all women should give birth at the clinic under the supervision of a trained and skilled health staff such as a nurse, midwife or doctor. Moreover, the new policy stopped nurses at the clinic from providing TBAs with delivery packs and other supplies. With the change in policy, TBAs were advised to work with headmen and other community health agents such as NHC members, CHWs, and safe motherhood action groups (SMAGs) to encourage pregnant women to attend ANC services and give birth at the clinic. In contrast, all the FGDs and IDI respondents with the exception of five 5 TBAs argued that many women in their communities still give birth at home, and that TBAs still conduct deliveries.

*“Yes, the new rule is there and many women know about it, the nurses tell them at the clinic. We also tell them during our community meetings. But we see that many of them still deliver at home” (36 year old headman).*

However, the five TBAs argued that, because of the new policy, they had stopped conducting deliveries in their communities. They explained that if called upon to assist a woman in labour at home, they would advise the family to find transport to take the woman to the clinic. Moreover, they explained that they accompanied the pregnant woman to the clinic. Furthermore, TBAs indicated that they only conducted deliveries at home in an “emergency situation” in order to prevent the woman from delivering on the way to the health centre.

Concerning the group of women who gave birth at home, respondents had mixed feelings. A quarter (25 %) of the FGD respondents (mainly the older mothers with six or more children) mentioned that both the older and younger women gave birth at home. They explained that, although most old women with many children gave birth at home, a large proportion of teenage girls also gave birth at home, because they were either not sure of the date of delivery or they did not inform their parents about the pregnancy, and thus, did not prepare for childbirth at the clinic.

In contrast, some FGD respondents (mainly those with one or two children) and all the IDI participants revealed that older women with many children were the ones who gave birth at home with the help of TBAs, mothers, neighbours, and friends. They explained that such women believed that they had enough experience with childbirth and knew whether they would develop complications or not. In contrast, they explained that young and inexperienced women and those who were at risk for complications gave birth at the clinic. Furthermore, those identified by nurses during ANC visits as being at risk of developing complications and those who had experienced complications during previous deliveries were more likely to give birth at the clinic. In addition, respondents explained that most women (both young and old) who were told that they had no complications during ANC (i.e., that the baby was lying well in the womb) ended up giving birth at home. They explained that, despite going for ANC at the clinic and being advised by nurses to give birth at the clinic, most old pregnant women with many children did not return.

### Theme 2: decision-making process regarding the place of delivery and use of traditional birth attendants

This theme focused on the decision-making process regarding home delivery and use of TBAs. Respondents had mixed feelings regarding women’s decision to give birth at home. They explained that families differed on who made the final decision. All the IDI respondents (except husbands) and most (70 %) old FGD respondents mentioned that the final decision whether the pregnant woman should go to deliver at the clinic or not was made by the husband.

“*The husband is the one who decides. We normally sit down with them to discuss, but they make the final decision. Some women decide alone” (36 year old mother).*

In contrast, all the four husbands and most (60 %) of the old FGD respondents with many children argued that women made the decisions themselves, based on their past experience with childbirth and whether they had enough money to buy things that were needed at the clinic. They only informed the husband about it.

Most young FGD respondents and some of the old FGD respondents (with few children) argued that young women and those without experience in childbirth consulted their parents for the place of delivery. Moreover, most young mothers mentioned that they followed advice from the nurses at the clinic.

Regarding the decision to seek the services of the TBAs, all FGD and IDI respondents mentioned that when women went in labour at home, they would inform their husbands to call the TBA to come home. If the husband was not home, the woman would ask her children, neighbours or parents to call the TBA.

*“When a woman is not feeling well, she sends her husband to go and call the TBA so that she assists her. If her husband is not there, she sends her children or her neighbours” (35 year old FGD participant).*

### Theme 3: reasons for home delivery and use of traditional birth attendants

This theme focused on the reasons for home delivery and use of TBAs. Our findings show that various personal, family and health-related factors as well as social and cultural norms played an important role in influencing women to give birth at home and to seek the services of TBAs.

#### Personal factors

##### Low risk perception

Low risk perception regarding their personal susceptibility to pregnancy and labour complications was one of the main reasons why most old women with many children delivered at home.

Most respondents explained that although most old mothers were aware of the severity of labour complications, most of them believed that, compared to other [younger] women, their personal susceptibility to pregnancy and labour complications was low and that they were not personally at risk of developing such complications, because of their experience with childbirth. Moreover, most (60 %) old FGD respondents (both those who had delivered at the clinic and those who had delivered at home) believed that they “knew themselves” pretty well because they had many successful deliveries in the past. In addition, they believed that they had enough experience giving birth and that they were able to recognise any possible complications that might arise during labour.

In contrast, most (80 %) young FGD respondents and a few old ones (mainly those who had given birth at the clinic), and all the midwives and NHC members explained that most young women delivered at the clinic because they had no experience giving birth and that they were scared of labour complications if they gave birth at home.

*“Most women with many children do not even worry about developing labour complications because they believe that they are used to it, and that they know themselves that they do not face problems when giving birth. They say that even if I experience problems, they will call the TBA or give me some herbs to drink; and then I will deliver” (20 year old FGD respondent/mother).*

#### Negative attitude towards nurses

All IDI respondents (except nurses) mentioned that most women who gave birth at home had a negative attitude towards nurses and the healthcare services because of the way they were treated during ANC care or during the previous deliveries at the clinic. In confirmation, most (80 %) old FGD, a quarter (25 %) of the young FGD respondents and most IDI respondents including all the mothers, NHC members and CHWs mentioned that nurses at the clinic were harsh to the women in labour and used abusive language to them.

Regarding their experience during childbirth at the clinic, women had mixed feelings. Five women including four (4) old FGD respondents, one (1) young FGD respondent with two children, and one (1) woman with one child participating in the IDI, who had given birth at the clinic during their previous deliveries, explained that nurses cared for them and saved their lives after they developed labour complications such as excessive bleeding, retained placenta and eclampsia (high blood pressure, severe headache and fitting). In contrast, twenty (20) out of the fifty (50) old FGD respondents (40 %) who had delivered at the clinic complained that during their previous deliveries at the clinic, nurses either shouted at them, left them to struggle alone in labour or did not assist them and their babies after labour.

Strikingly, all the husbands had a positive attitude towards nurses, whereas TBAs avoided commenting on the issue. Nurses explained that most clinics did not have enough staff to attend to the women in labour and those with general medical conditions.

#### Family-related factors

Dependence on the husband for financial support and decision-making was seen as one of the main reasons preventing women from giving birth at the clinic. The husband was perceived as the most important person in the decision-making process and his decision was usually final and most pregnant women would accept it.

All IDI respondents (except husbands) and most FGD respondents (both old and young) mentioned that pregnant women depended on their husbands as the provider. If the husband did not have enough money to provide for his wife, he would either delay making the decision to allow the wife to go to the clinic or stop her all together. In this case, she would deliver at home.

In contrast, all the husbands participating in the IDIs argued that men in their communities did not stop their wives from delivering at the clinic. Rather, they encouraged and supported them to do so.

*“Most women fail to go to deliver at the clinic because of their husbands. They depend on their husbands to allow them” (43 year IDI participant/NHC member).*

#### Health system-related factors

All the FGD respondents and all the IDI participants indicated that most women gave birth at home because they perceived various barriers preventing them from delivering at the clinic. The main barriers cited included lack of funds for baby clothes and requirements for the mother during and after labour, poor quality of services at the clinic due to non availability of nurses, negative experiences with nurses during ANC visits or delivery during their previous pregnancies, long distances to the clinic and high transport costs, poor state of labour wards and absence of maternity waiting homes (MWHs), where it was present, it was in deplorable state. Other barriers included lack of funds for food for the pregnant woman while waiting for labour at the MWH.

In contrast, all IDI respondents (except husbands) argued that some families did not prepare for childbirth and that many husbands did not support their wives to find resources to use when staying at the clinic, waiting for delivery.

#### Social and cultural norms

All the IDI respondents (except husbands), and some (40 %) of the FGDs respondents (both young and old with few children) mentioned that, especially old women with many children delivered at home because they believed that they should not be delivered by either a young nurse or a male staff at the clinic. They explained that most women were aware of the kind of nurses they would meet at the clinic − a young or old nurse, or a male one − and should there be a young or male nurse, some women would stay away and deliver at home, assisted by TBAs who were female and could be about their own age or older. In addition, young teenage and inexperienced women’s delivered at home accidentally because they were inexperienced with childbirth and did not know when they would go into labour.

### Reasons for seeking the assistance of the TBAs

Our findings show that pregnant women’s positive attitude towards TBAs was one of the main reasons why most of them preferred the services provided by TBAs.

#### Trust in traditional birth attendants

One of the main reasons for seeking the services of the TBAs was the trust women had in them. All IDIs respondents (except nurses and TBAs) and most (70 %) FGD respondents (mainly old mothers) explained that women were happy with TBAs because they were always and immediately available when they were called upon to assist the woman in labour. They explained that, in contrast with nurses who were perceived as being absent from the clinics, TBAs would rush to the woman’s home to assist as soon as they received the message, regardless of the time of the day or weather conditions. This trust in the TBAs’ availability made most old women choose to stay at home rather than travel to the clinic where they would not find the help they needed.

Moreover, all the IDI respondents (except nurses and husbands) and 60 % of the FGD respondents (both young and old respondents) explained that in some instances, women who decided to go to the clinic to deliver did not find nurses to assist them. Rather, they ended up being assisted by the accompanying relatives or call the TBA from the community to assist them; some women ended up going back home and call the TBAs to assist them.

*“If you are not feeling well, you just send someone to call the traditional birth attendant to come and deliver you; she will come immediately and wait until you deliver. Whatever time you call her….even if it is raining, even at night she comes to help you” (36 year old mother).*

n contrast, all the TBAs explained that, although they were available when called upon, they were overwhelmed with work. Similarly, most IDI respondents including headmen, husbands, and CHWs, and old FGD respondents indicated that, TBAs were few in the community, and they often had to walk long distances when called upon by women. They explained that if TBAs were not available, women received help from their parents, mothers-in-law or neighbours.

When probed why TBAs were so few, respondents gave varying answers. All the TBAs, NHC members, CHWs and headmen mentioned that some of the trained TBAs had left their villages and that they were not replaced due to the lack of training programmes stemming from the new health policy. In addition, respondents explained that some TBAs decided to become inactive because they never received any incentives or logistical support from the Ministry of Health. In addition, respondents explained that some TBAs decided to become inactive because they never received any incentives or logistical support from the Ministry of Health.

*“We were trained by the district health office a long time ago, but now they have stopped training us. This has caused problems because we are now few. Like me I am alone here; I have to assist even women from the other villages because some TBAs have left their villages.” (40 year old TBA).*

#### Familiarity with traditional birth attendants

In addition to the trust in the TBAs, being familiar with TBAs was seen as one of the main reasons why women decided to use their services. Most IDI respondents including all the headmen, CHWs and NHC members and FGD respondents (especially the old women who had many children and had given birth at home) explained that women felt free with TBAs because they lived with them in the community − unlike nurses who were total strangers and sometimes shouted at the woman in labour. Moreover, women were free to discuss the progress of labour with the TBA and to inform her about how they felt about it − whether they needed food, water, or some rest. If a woman was not cooperative enough during labour, the TBA would not shout at her. Rather, she would encourage the woman to “push” so that the baby would not suffocate and die in the process. Respondents added that if a TBA noticed they were getting tired, she would ask them to “take a rest” and would either give some light porridge to eat or some *chibwantu (*local non- alcoholic drink) to drink. Moreover, women were also happy with the way TBA cared for them and their babies during and after giving birth.

*“Most of us are free with the TBAs because we know them and we live with them. We are used to them. But for the nurses you find that you don’t even know each other (42 year old mother).*

In contrast, most young FGD participants argued that some TBAs were harsh to the women in labour, and that they did not allow mothers to decide to go to the clinic in case of complications. Moreover, these respondents together with most IDI respondents (including nurses, CHWs, NHC members and three out of the four husbands interviewed) mentioned that TBAs did not transfer women with complications to the clinic in time.

#### Confidence in skills of traditional birth attendants

In general, most (70 %) old FGD respondents (comprising older mothers) believed that TBAs had enough skills and experience to assist a woman during labour, child birth and that should labour complications occur, TBAs had enough skills to recognise them on time and refer the mother to the clinic. The main skills TBAs were believed to possess included: 1) carrying out an abdominal examination to assess the position and status of the baby during ANC; 2) carrying out an abdominal and vaginal examination to assess the stage and progress of labour; 3) cleansing the perineum to avoid infections during labour; 4) conducting the actual delivery, including cutting the cord and management of the third stage (delivery of the placenta) to prevent post partum haemorrhage; 5) detecting danger signs or complications, and deciding/making a referral to the clinic. Furthermore, TBAs were believed to have skills to advise the mothers on cord care. They could assess if the cord stump was infected, and refer the baby to the clinic for medical attention.

Moreover, most old FGD respondents (both those who had given birth at home and at the clinic) explained that most old mothers had confidence in the skills of the TBAs because they had seen TBAs providing ANC services and conducting labour at the clinic, and that nurses had allowed them to do so. Some of the mothers had actually been attended to by TBAs during their previous delivery at the clinic. Thus, women with such an experience were reinforced in their beliefs about the TBAs’ skills, and were more likely to decide to give birth at home in future pregnancies, and consult the TBAs for assistance.

*“These same TBAs are the same ones who help us even at the clinic. They are able to assist us during labour and if you face difficulties she can examine you to see if the baby is alive. They are better than some nurses who refuse to help us because they say they are not trained in midwifery” (36 year old mother).*

In contrast, all the IDI respondents and most young FGDs respondents and a few (25 %) older mothers (who had given birth at the clinic during their previous pregnancies) argued that, despite their experience, some TBAs were not skilled or trained enough to handle labour complications at home. They explained that TBAs no longer received any logistical support from the health centres, and that they had no instruments or supplies to use in case of complications such as severe bleeding or eclampsia. Moreover, TBAs were believed to have no skills to recognise pregnancy and labour complications, and that sometimes they delayed referral of the woman to the clinic because they did not know when to do so. Furthermore, some young FGD respondents complained that TBAs delayed sending women to the clinic because they expected to receive an incentive in form of a chicken or an agreed-upon amount of money after conducting a successful delivery.

Moreover, IDI respondents including all the headmen, nurses and NHC members and most FGD respondents who had given birth at the clinic (both young and old) believed that, in case of labour complications, TBAs had no access to the referral services such as a reliable and readily available community transport to the clinic. In addition, they had no access to communication facilities such as good road network or mobile phone services to call for help and transfer the woman to the clinic or the district hospital in good time for further management.

Nevertheless, headmen, NHC members and CHWs, and most FGD respondents (both young and old) who had given birth at the clinic complained that many clinics in their communities had no ambulances. Most clinic staff relied on the ambulance from the district hospital which took long to arrive at the health centre when needed. Moreover, women with labour complications were transferred for long distances on bad roads to reach the district hospital for specialist care.

## Discussion

In the current study we set out to investigate the reasons why women would decide to give birth at home rather than at a clinic, and how they viewed TBAs. To this end, we interviewed women and other key informants from the area, i.e. husbands, mothers, TBAs, headmen, NHC members, CHWs, and health workers in Kalomo, Zambia.

Consistent with previous studies [10–12], our findings suggest that most women in Kalomo district are aware of the presence of TBAs in the area and have a positive attitude towards them. They believe that TBAs play an important role in providing MHS to the women in the community including health education, ANC services and delivering services. Moreover, our findings show that, despite the policy change in Zambia stopping the training of TBAs and recommending that TBAs stop conducting deliveries at home, and that all women should give birth at a health facility under the skilled supervision of a qualified health personal such as a nurse, midwife or a doctor, many women still give birth at home and TBAs still play an important role in assisting them. These findings are consistent with our previous studies [10, 11] and studies from other developing countries [17, 20] which reported that home delivery and use of TBAs were still being preferable for some women in rural areas. Our findings also suggest that, despite the need to have all women give birth under skilled care, public health strategies and policies need to take into consideration women who have no access to facility-based skilled delivery services in rural areas, and the consequences of denying them the very basic delivery services and benefits from a trained TBA. For example, lack of medical and logistical supplies to enable TBAs conduct clean deliveries may predispose women and their babies to the risk of infection during labour. Our findings provide important evidence for the debate in Zambia and many developing countries about whether the current policy on use of TBAs needs to change or not and suggest benefits to be gained from public health strategies involving TBAs as important partners in improving MHS in remote areas where their services are highly utilised.

Regarding the decision-making process on the place of delivery and use of TBAs, our study highlights important findings. Although most respondents believed that husbands are the main decision-makers, the four husbands in our sample denied making the final decisions for their wives; they believed that women make the decision and only inform the husband about it. Moreover, especially young couples consult their parents for the final decision. Furthermore, when considering calling for the services of the TBAs, women decide when the TBAs should be called home. When in labour, the woman will normally ask the husband to call the TBAs to come home and assist her. In the absence of the husband, children or neighbours will assist in calling the TBAs. This finding is important as it is in contrast with previous studies [10, 21–23], which suggested that husbands make the final decision whether the woman should deliver at home or not. Our finding suggests that pregnant women are also active participants in the decision-making process regarding their health seeking behaviour, especially when it comes to the use of TBAs.

Consistent with previous studies [10, 11, 15–17], our findings show that various factors including individual, family-related, socio-cultural norms and health system-related factors influence women’s decisions to give birth at home and seek the services of the TBAs. For example, our findings suggest that despite being aware about the risks associated with pregnancy and child birth, as well as their severity, and the risks involved in home delivery without a skilled birth attendant, most women do not believe that they are personally susceptible to the complications associated with childbirth and home delivery. This finding is consistent with other studies [23, 24] which have shown the importance of perceived susceptibility as an important factor influencing health behaviour change. Interestingly, this finding is in contrast with earlier studies in Zambia as well as in other developing countries [25–27] which suggested that women who gave birth at home lacked knowledge about the risks associated with this behaviour. Moreover, persuasive messages which focus on building knowledge and creating awareness regarding the severity of health problems might not prove successful if people under-estimate their personal risk and susceptibility [23, 24].

In addition to low risk perceptions, our findings suggest that most women who gave birth at home had a negative attitude towards the health services provided at the clinic because of their perceived poor quality due to non availability of nurses, negative experiences with nurses during ANC visits or delivery during their previous pregnancies, poor state of labour wards, and absence of MWHs. Moreover, women perceived various barriers including lack of funds for baby clothes and requirements for the mother during and after labour, long distances to the clinic and high transport costs which prevented them from delivering at the clinic. In our opinion, public health interventions focusing on improving maternal health outcomes would benefit from targeting women’s perceived susceptibility as an important determinant of their health behaviour change. Public health interventions would also benefit from mitigating physical and economic barriers preventing women from accessing maternal healthcare services.

Our findings show that social and cultural norms promoting women to depend on their husbands, parents and important others for the final decision about the place of delivery contribute to most women giving birth at home. For example, our findings suggest that despite participating in the discussion regarding preparations for childbirth, most women depended on their husbands and parents for the final decision. Moreover, the low social status of women and their dependence on their husbands for financial resources and support might be causing a delay in decision-making about the place of delivery which often resulted in most women giving birth at home. These findings are consistent with our previous findings [10, 11] and those from Ghana [23] and Bangladesh [28]. These studies highlighted the important role decision-making and women’s autonomy play in the use of facility delivery services and showed that women who had more autonomy were more likely to deliver in a health facility. In addition, these findings are consistent with those by Thaddeus and Maine [15] which showed the importance of decision-making in limiting access to, and utilisation of, maternal healthcare services. Together, these findings highlight the importance of empowering women with decision-making skills and resources in order to mitigate barriers that make it difficult to do so. These findings also highlight husbands, parents, and friends as important targets for interventions.

Our findings also suggest that women’s positive attitude towards TBAs is an important factors motivating women to give birth at home. In general, respondents indicated great trust and confidence in TBAs and held many positive beliefs about them and the benefits to be gained from using their services. They described the TBAs as available, reliable, familiar, skilled, polite, patient, respectful and caring.

Consistent with other studies [7, 10–12, 17], living with the TBAs in the same community, knowing them, and having trust in them were found to be important factors influencing women’s attitude towards TBAs. Most women indicated that TBAs had a “more humane” attitude towards mothers during labour than the nurses. Indeed, the nurses, who, could usually not be found at the clinic, shouted at them during delivery. This finding is consistent with previous studies [10, 11, 17, 27, 29] which have suggested that public health interventions would benefit from focusing on improving the staffing and motivation levels for midwives and nurses in the clinics, as well as encouraging collaboration with TBAs.

Women’s decisions on the place of delivery seem to be influenced by their evaluation of the comparative advantages of either delivering at the clinic or staying at home waiting for assistance from the TBA. Furthermore, women’s evaluation of these perceived benefits seems, to a large extent, to be based on their past experience with either delivery at the clinic or at home. Upon engaging in health behaviour, women will evaluate the expected outcomes, based on the available information their past experience [10, 11, 17]. Thus, public health interventions and formal health systems would benefit from recruiting and motivating midwives and nurses to serve in their local communities, and providing traditional birth attendants with requisite skills. Indeed, incorporating the traditional birth attendants in the formal health system might significantly improve the number of institutional deliveries.

Interestingly, giving birth in one’s house was associated with various gains to the woman − such as privacy and comfort during labour, as well as maintaining a connection with the “rest of the family”. These perceived benefits of giving birth at home were contrasted with the hardships of sleeping in the mothers’ shelter without beds or mattresses and giving birth in a clinic labour ward where they perceived no privacy. Thus, the women believed that not all pregnancies should be medicalised. Rather, low- risk pregnancies should be identified during ANC and be allowed to take place at home under supervised and skilled care linked to a functioning referral system. This finding is consistent with studies from Nigeria [30], but also from other developed countries [27, 31]. For example, deJonge et al. in The Netherlands [31] − a country in which around 21 % of pregnant women choose to give birth at home [32] − showed that planned home birth for low risk women was not associated with an increased risk of adverse maternal outcomes.

In the current study, women’s evaluation of their and their baby’s perceived medical safety was not only based on the availability of the TBAs during and after labour, but also on the woman’s perception of the birth attendants’ skills. This finding is in line with previous studies [10, 11, 21, 22, 33, 34] which showed the importance of perceived quality of care in influencing women’s attitude towards the place of delivery and the birth attendant. Future studies should focus on the evaluation of the perceived and actual quality of care among TBAs and evaluate their level of skills. Such an evaluation could have important policy implications for the continued use of TBAs in obstetric care in developing countries, and the decision whether to provide formal training to this group anymore.

Some potential limitations of our study should be noted. First, these findings are only based on the experiences of the women who accepted to participate in the FGD and a few IDI respondents; since the recruitment of the FGD respondents was done in the community during community meetings, we do not have information on how many were approached or how many declined participating in the study. Moreover, the experiences of the husbands were not explored due to logistical reasons. FGDs with husbands could have provided balanced views on the factors that determine the choice of delivery places and birth attendant preferences.

## Conclusion

To conclude, our findings show that most women give birth at home due to several individual, family and health system-related factors including women’s low risk perception regarding their personal susceptibility to labour complications, negative attitude towards facility-based delivery services, lack of decision- making autonomy regarding child birth, and dependence on the husband and other family members for the decision regarding the place of birth. Moreover, various physical and socioeconomic barriers including long distances, lack of money for transport and the requirement to bring baby clothes and food while staying at the clinic prevented women from delivering at the clinic.

Women’s positive attitude towards TBAs, e.g., women’s perception that TBAs were skilled, respectful, friendly, trustworthy and available when they needed them, motivated them to seek the TBAs’ services and prevented them from utilising skilled facility based delivery services.

These results offer starting points for future interventions which, in our opinion, should focus on improving women’s decision-making autonomy regarding childbirth and also empowering them with skills and resources to improve their socioeconomic status. These findings also highlight the need to help women perceive the benefits of delivering at the clinic − which they currently often do not recognise. Moreover, interventions and policy should focus on husbands, parents, and friends as well as on improving staffing levels in the clinics, e.g., by making sure nurses and midwives are always available, and by motivating midwives and nurses to serve in their local communities by providing them with financial, housing and training incentives. The linkages between TBAs and midwives should be strengthened through close collaboration and establishment of a functional referral system.

Moreover, although, due to a policy change the training of TBAs to acquire basic midwifery skills does not receive funding anymore [13], the current study suggests that maternal health outcomes might improve if this medical training were reinstated, especially, TBAs themselves also stated they were often lacking in the required skills. Finally, interventions aimed at mitigating the physical and economic barriers through providing new mothers’ shelters and refurbishing existing ones, and providing women with the requisite resources such as mother-baby packs, might persuade more women to go to a health centre and wait for delivery there.
